# Systematized Nomenclature of Medicine–Clinical Terminology (SNOMED CT) Clinical Use Cases in the Context of Electronic Health Record Systems: Systematic Literature Review

**DOI:** 10.2196/43750

**Published:** 2023-02-06

**Authors:** Riikka Vuokko, Anne Vakkuri, Sari Palojoki

**Affiliations:** 1 Unit for Digitalization and Management Ministry of Social Affairs and Health Helsinki Finland; 2 Perioperative, Intensive Care and Pain Medicine Helsinki University Hospital Vantaa Finland; 3 Unit for Digital Transformation European Centre for Disease Prevention and Control Stockholm Sweden

**Keywords:** clinical, electronic health record, EHR, review method, literature review, SNOMED CT, Systematized Nomenclature for Medicine, use case, terminology, terminologies, SNOMED

## Abstract

**Background:**

The Systematized Medical Nomenclature for Medicine–Clinical Terminology (SNOMED CT) is a clinical terminology system that provides a standardized and scientifically validated way of representing clinical information captured by clinicians. It can be integrated into electronic health records (EHRs) to increase the possibilities for effective data use and ensure a better quality of documentation that supports continuity of care, thus enabling better quality in the care process. Even though SNOMED CT consists of extensively studied clinical terminology, previous research has repeatedly documented a lack of scientific evidence for SNOMED CT in the form of reported clinical use cases in electronic health record systems.

**Objective:**

The aim of this study was to explore evidence in previous literature reviews of clinical use cases of SNOMED CT integrated into EHR systems or other clinical applications during the last 5 years of continued development. The study sought to identify the main clinical use purposes, use phases, and key clinical benefits documented in SNOMED CT use cases.

**Methods:**

The Cochrane review protocol was applied for the study design. The application of the protocol was modified step-by-step to fit the research problem by first defining the search strategy, identifying the articles for the review by isolating the exclusion and inclusion criteria for assessing the search results, and lastly, evaluating and summarizing the review results.

**Results:**

In total, 17 research articles illustrating SNOMED CT clinical use cases were reviewed. The use purpose of SNOMED CT was documented in all the articles, with the terminology as a standard in EHR being the most common (8/17). The clinical use phase was documented in all the articles. The most common category of use phases was SNOMED CT in development (6/17). Core benefits achieved by applying SNOMED CT in a clinical context were identified by the researchers. These were related to terminology use outcomes, that is, to data quality in general or to enabling a consistent way of indexing, storing, retrieving, and aggregating clinical data (8/17). Additional benefits were linked to the productivity of coding or to advances in the quality and continuity of care.

**Conclusions:**

While the SNOMED CT use categories were well supported by previous research, this review demonstrates that further systematic research on clinical use cases is needed to promote the scalability of the review results. To achieve the best out-of-use case reports, more emphasis is suggested on describing the contextual factors, such as the electronic health care system and the use of previous frameworks to enable comparability of results. A lesson to be drawn from our study is that SNOMED CT is essential for structuring clinical data; however, research is needed to gather more evidence of how SNOMED CT benefits clinical care and patient safety.

## Introduction

### Background

The Systematized Medical Nomenclature for Medicine–Clinical Terminology (SNOMED CT) is an extensive, multi-hierarchical clinical terminology system. It provides a standardized and scientifically validated way of representing clinical information [1]. The application possibilities of SNOMED CT are well documented [1-5], and various guides describe the following types of implementation: clinical records, knowledge representation, data aggregation, and analysis. Specifically, the previous literature describes the various development goals of SNOMED CT. For example, SNOMED CT can be used as a standard for electronic health records (EHRs) for classifying or coding clinical information. Additionally, standardized terminology advances data indexing, storing, and retrieving. This supports sharing of patient information across medical domains and organizations in ways that promote continuity of care. As a large-scale terminology system, SNOMED CT also enables knowledge representations in clinical guidelines and care pathways, which can be used, for example, with decision support [2-5].

Data recorded in EHRs are primarily used to provide care to patients. The potential of SNOMED CT to improve data quality and facilitate interoperability, and thus improve patient safety, has long been noted in existing research. Studies have shown that structured and standardized EHRs also increase data reuse possibilities [6]. The European Union and the US Healthcare Information Technology Standards Panel have noted possibilities provided by SNOMED CT and taken steps toward increasing semantic interoperability, reuse, and the exchange of health data. Data recorded in local systems can also be used to support the achievement of broad health policy goals. The importance of SNOMED CT is expected to gradually grow, but at the same time, there is a need to tackle the complex implementation challenges that may arise [1,7,8].

When implemented in EHRs, SNOMED CT is used to represent clinical information consistently and comprehensively [1,2]. Despite the widespread adoption of EHRs that are certified to follow terminology standards, and although SNOMED CT is used in more than 50 countries, there are only a few published reviews about its clinical use. Most studies have focused on theory and predevelopment or design [1,2,8,9]. Moreover, studies in the past have analyzed general factors related to EHR adoption but have not explored the factors associated with less advanced EHR product implementations as compared to more advanced and mature EHR systems. In the context of SNOMED CT implementation, the maturity of an EHR system is a relevant factor to appropriately maximize the benefit from previous experiences [10-12].

In summary, even though SNOMED CT has been extensively studied as a clinical terminology system, previous research has repeatedly documented a lack of detailed evidence for SNOMED CT in clinical use cases [2,3]. Considering that implementing SNOMED CT is a challenging proposition [2], the identification of specific barriers and facilitators to implementing SNOMED CT in clinical use is of paramount importance to further promote its adoption. Evidence from use cases might support implementation and provide guidance on avoiding deployment pitfalls. Therefore, we aimed to explore the available evidence in previous literature reviews of clinical use cases of SNOMED CT integrated into EHR systems or clinical applications during the last 5 years of continued development [2,3,5,13].

### Objectives

The aim of this review is to provide an overview of published studies on SNOMED CT clinical use cases in the context of EHRs. In this study, we apply categories from previous research for analyzing use purpose and use phase for the terminology. Moreover, we present core benefits by summarizing the observations from the EHR use cases [3,5,13].

Our research questions are as follows: (1) What are the main clinical use purposes of SNOMED CT during the last 5 years of development? (2) What kinds of use phases of SNOMED CT are identified in these studies? and (3) What are the summarized clinical benefits documented in each SNOMED CT use case?

## Methods

To explore EHR-related SNOMED CT use cases in recent research, our research team set out to conduct a systematic literature review. Our team consisted of a medical expert with decades of experience in clinical care and two health and medical informatics experts. To analyze EHR use cases where SNOMED CT terminology was applied, we extended the concept of EHR systems to cover EHR-related applications and software in clinical use. The word “clinical” refers to “medical work or teaching that relates to the examination and treatment of ill people” [14]. In our review, a use case consisted of SNOMED CT integrated into an EHR system in various stages of use, either in preuse development or in design, piloting, testing, implementation, use, or postimplementation evaluation [15].

The team followed the Cochrane review protocol [16] to plan the necessary steps for this study design (Multimedia Appendix 1). Within the team, the application of the protocol was modified step-by-step to fit the research problem by first defining the search strategy, identifying the articles for the review by isolating the exclusion and inclusion criteria for assessing the search results, and lastly evaluating and summarizing the review results.

**Figure 1:**
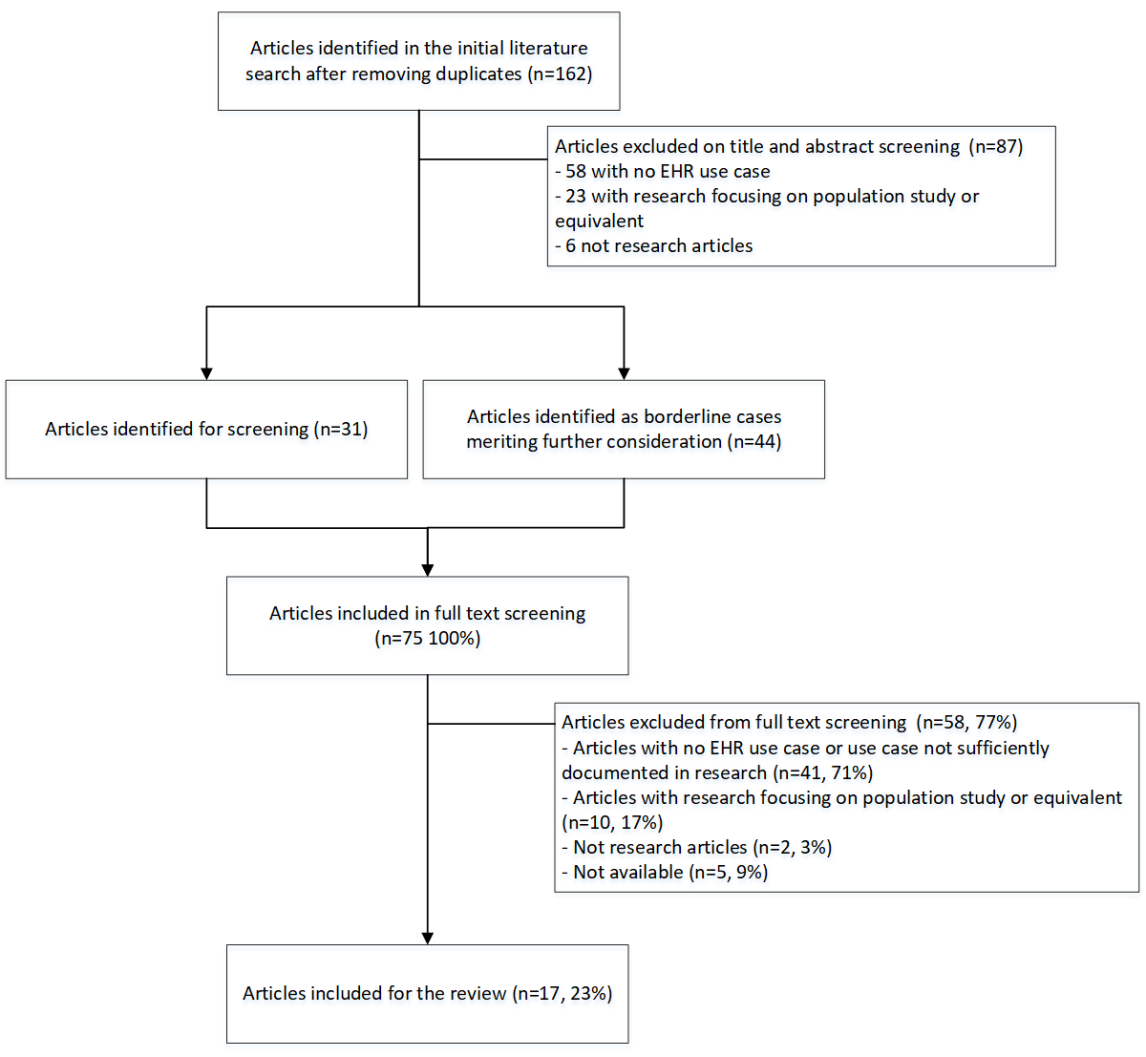
Application of the review protocol as a flowchart. EHR: electronic health record.

We defined our search strategy by adding variations of search terms and testing the suitability against the search results. A search with key words “EHR,” “EMR,” “electronic health record,” or “electronic health record system” produced a large number of search results. Combining these terms with “SNOMED CT” produced relevant search results for our review purposes. Furthermore, adding filters (Textbox 1) did not cause a significant change in the search results. A search of PubMed using the systematic-review methods filter was undertaken in March 2022 and resulted in 162 original articles after removing duplicates (Figure 1). Our results cover the last 5 years of research; thus, our review forms a continuation of previous reviews [2-5].

**Search strategy and filters used.**

**Search terms**

(((((Ehr) OR (Emr)) OR (electronic health record)) OR (electronic health record system)) OR (electronic medical record) AND ((fha[Filter]) AND (fft[Filter]) AND (2016/1/1:2022[pdat]))) AND ((Snomed ct) OR (snomed CT) AND ((fha[Filter]) AND (fft[Filter]) AND (2016/1/1:2022[pdat]) AND (english[Filter])))

**Filters**

Abstract, Full text, English, Abstract, Full text, English, from 2016/1/1 – 2022

The exclusion and inclusion criteria were defined before conducting the search. We based our criteria on our research questions and previous research. We defined the exclusion criteria as follows: First, the original article had to document an EHR use case where SNOMED CT was being tested, piloted, implemented, or used in a clinical context. This excluded, for example, research concentrating on theoretical building, evaluation, or validation of SNOMED CT. Second, we excluded population studies and, for example, cohort studies where SNOMED CT was used to define, extract, and harmonize study data and where no EHR-related design or use goals were documented. Third, we excluded editorials, posters, and other such sources to limit the review to original research articles. While reading the articles, we discussed how well the exclusion criteria corresponded to the delimitation made based on our research questions and the conclusions we arrived at while reading the research content as presented in the original articles.

After the first exclusion based on article headings and abstracts, we had excluded 87 articles. We included 31 articles that seemed to relate to our research question. Moreover, 44 borderline cases merited special consideration to determine if they should be excluded or included (Figure 1). The two researchers set out to read a total of 75 full-text articles. The information extraction and documentation template for final inclusion had been defined based on previous research [3,5,13] and research team agreement (Table 1). During the final reading, the documentation template for information extraction was concurrently refined.

Figure 1 illustrates how the search results were analyzed during exclusion and inclusion screening. In the end, we included 17 original articles in the review analysis. Our final inclusion was confirmed by the research documentation that illustrated the SNOMED CT use case in an EHR or EHR-related application and software in clinical use.

**Table 1:** Criteria to categorize Systematized Nomenclature of Medicine–Clinical Terminology use in the review.

Criteria	Definition
Clinical use context	Refers to clinical domain or specialty as documented in the study.
EHR^a^ system	Refers to EHR systems or other EHR-related applications or software as documented in the study.
Users	Refers to intended users of the SNOMED CT^b^ integrated into the EHR as documented in the study.
SNOMED CT use category	Refers to primary purpose for using SNOMED CT as documented in the study [3,5,13]. Based on research team agreement, the following categories were used: standard for EHR or for a clinical application, retrieval or analysis of patient data, data extraction (used to classify or code in a study), proving merit of SNOMED CT, and development of automated coding.
SNOMED CT use phase	Refers to the stage of the SNOMED CT use as documented in the study [3,5]. The stages used by the research team were “in development,” “in pilot,” “in implementation,” “in use,” and “after implementation [or in use] evaluation” (ie, proof of merit). Thus, for example, theoretical research was excluded.
SNOMED CT core benefits	Refers to research team’s summary of which areas of identified benefits the research added value to, if available [3,5]. The categories were “improving quality of care and patient safety”; “improving continuity of care”; “enabling a consistent way of indexing, storing, retrieving, and aggregating clinical data”; “improving data quality”; and “improving coding productivity.”

^a^EHR: electronic health record.

^b^SNOMED CT: Systematized Nomenclature of Medicine–Clinical Terminology.

## Results

### Characteristics of the Publications

In total, we analyzed 17 articles [17-33]. The earliest research selected for the final review was published in June 2017 and the last in February 2022. On an annual basis, the largest number of articles was published as late as 2021 (6/17). The country of publication was identified for all publications. The country that published the highest number of articles was the United Kingdom (5/17), followed by the United States (3/17), Australia (2/17), and Spain (2/17). Canada, Denmark, Switzerland, the Republic of Korea, and Germany each accounted for one use case. The selected articles were published in 13 different peer-reviewed journals. The characteristics of the publications are summarized in Multimedia Appendix 2.

### Contextual Factors of the Clinical Use Cases

To verify the appropriateness of the use cases in regard to our research questions, the clinical context was described for 14 articles. In the remaining 3 articles (3/17), the clinical context was not named, but the research account otherwise described the clinical use case in relation to EHR use. With respect to specialties, 2 cases were from neurology, and 1 case each from pulmonology (asthma), cardiology, oncology, general medicine, pediatrics, and rare diseases. One of the cases did not describe an exact specialty but concerned the prehospital unit in emergency care, and one concerned an outpatient clinic. Four cases mentioned either primary care or tertiary care.

The EHRs were poorly described in most of the articles, and specific descriptions of the EHRs did not follow any uniform structure. Thus, the nature of the results is descriptive. Only one of the publications named the exact product. That study concerned a comprehensive hospital information system, a high-maturity EHR with tools for functions such as supporting care coordination and continuity of care. One of the systems was described as a prehospital patient record that was not integrated into the hospital EHR. One system used by general practitioners consisted of integrated software for clinical use, and one was described as a primary care EHR. Six systems (6/17) were hospital EHRs. Among the system types were also the following: an “outpatient and inpatient EHR,” a “centralized EHR with web-interface,” and a “local EHR.” One of the use cases described the system generally as an “ehr.”

To further verify the clinical orientation of the use cases, we analyzed the professional groups involved in each of them. Although users were described, it was not clearly stated which professional groups were the intended users of SNOMED CT (eg, nursing informatics, medical informatics, or multi-professional users). Seven of the use cases (7/17) described the users in an exact way. Four cases (4/17) were applied by physicians, 2 (2/17) by nurses, and 1 by physicians and nurses. Two of the cases (2/17) were applied by a multi-professional team of clinicians, clinical and medical informatics professionals, clinical domain experts, terminologists, and clinical coders. Two of the articles specifically concerned clinical coders. Two of the remaining cases were generally described as having been applied by “clinicians.” Four of the cases (4/17) concerned researchers themselves, for whom specific clinical backgrounds were not reported. Contextual factors are presented in Multimedia Appendix 3.

### SNOMED CT Use Purpose

All 17 articles described one or several use purposes for the terminology. Implementing SNOMED CT as a standard terminology in the EHR was typically grounded on clinical needs for standardizing patient information. Here, the SNOMED CT use purpose refers specifically to the primary use goal of the terminology as integrated or being implemented into an EHR. The most common category of use purpose (8/17) was SNOMED CT adopted as a common standard for EHRs. An additional 2 studies (2/17) described the goal of implementing SNOMED CT as a standard in a separate clinical application integrated in the EHR system. The use cases described communication and coordination needs, such as between hospital units or between inpatient and outpatient care, with the goal of promoting more reliable continuity of care and ultimately a higher quality of care. Accurate and timely diagnosis information with SNOMED CT deployment was reported as a crucial clinical need since it is major information in patient care. In 2 of the studies, SNOMED CT was implemented into the documentation of the problem list to increase the usefulness of the patient information and to organize the problem list content. Additionally, SNOMED CT was used to ensure effective data migration between systems.

The primary use purpose described in 2 articles was to retrieve or analyze patient data for clinical research. This enhanced data retrieval, analysis, and sharing for clinical research across multiple hospitals. Multisite data sharing and distributed analysis was supported by common terminology and by common data models. These 2 use cases utilized a medical annotation toolkit that included a web interface for extracting needed concepts. An additional 2 studies focused on data extraction, where SNOMED CT was used to classify and code patient data for research purposes. The use purpose in these 2 studies was building clinical pathways and patient selection criteria based on terminology coding. Natural language processing of clinical, pathology, and genomics data was used for further clinical research. Moreover, the use cases illustrated the challenges of data sharing between inpatient care and a virtual hospital visit.

Two of the original articles described, to a degree, the already established use of SNOMED CT with a focus on proving the merit of the terminology use in a clinical setting. The reasons for poor clinical coding of patient data after 2 decades of EHR use are manifold; two main reasons are lack of motivation and training. Support tools for the interoperable recording of diagnostic, treatment, and interventional patient information can be advanced, for example, with domain-specific development. One of the articles documented automated clinical coding as the driving purpose for SNOMED CT development. The development of computer-assisted coding may, through careful review and validation, improve the productivity of clinical coders. Different classification systems, such as the International Classification of Diseases–10, are typically linked and mapped to SNOMED CT for suitability in clinical use. An overview of the SNOMED CT use purposes is provided in Multimedia Appendix 3.

### SNOMED CT Use Phase

The phase of use was identified in the literature with varying accuracy, which is why the research team discussed these categories during the analysis. Clinical use phase was documented in all 17 articles (100%), but in ambiguous ways. The most common category of use phases was SNOMED CT in development, which was documented in 6 articles (6/17). Development was described as an iterative process of analysis, validation, and standardization or building and mapping EHR-structured content that requires coordination and communication between stakeholders to improve the quality of care; as such, this was expected to be a process that could span several years.

SNOMED CT in use was identified as the use phase in 5 EHR-related use cases (5/17) and in implementation in 4 use cases (4/17). In the EHR-related use cases, SNOMED CT had been chosen as the base terminology system in the EHR or in a specific domain documented in the research to improve clinical information recording and coding, develop clinical pathways, and extract clinical data. The implementation cases addressed specifically improved the clinical recording of patient data by supporting clinicians’ language and semantic selection with SNOMED CT or with a combination of SNOMED CT and other classification or terminology systems. In addition, 1 article documented a pilot use of SNOMED CT in EHR use cases, and 2 articles described the merits of SNOMED CT use with a more proven merit approach or through after-implementation evaluation. The pilot case evaluated cases of missing or mislabeled clinical data with, for example, nonstandard concepts or use of abbreviations. The evaluation of SNOMED CT use aimed to determine what had been achieved with fully integrated EHR services in patient care and to evaluate the impact of using SNOMED CT to record clinical meanings. As additional benefits, the secondary use purpose for using patient information was mentioned. An overview of the SNOMED CT use phase is provided in Multimedia Appendix 3.

### Core Benefits of SNOMED CT

The research team identified and summarized core benefits of SNOMED CT as documented in the 17 use cases. The team categorized the benefits based on the previous literature (Multimedia Appendix 3). The core benefits were related to terminology use outcomes. The most common category was increased data quality, with 8 articles (8/17). Semantic-level core benefits were built on the scope and comprehensiveness of the terminology. In the use cases, SNOMED CT supported not only clinical meaning standardization but also the language of choice. For clinical use purposes, custom concept dictionaries or language-specific subsets were built for a chosen language. Further benefits of implementing SNOMED CT were 2-fold: the parallel development of EHR technology and standardization. One UK use case documented evidence of increased interface usability and user satisfaction by clinicians. However, clinicians reported that adopting a new approach for data recording was a gradual process requiring time.

Four articles (4/17) documented the benefits category of enabling a consistent way of indexing, storing, retrieving, and aggregating clinical data. One use case concentrated on the benefits for data retrieval. Documented benefits pointed to the documentation of clinical events with richer detail. The productivity of coding was the main benefit categorized in 1 use case, increased quality of care in 2 use cases, and increased continuity of care in 1 use case. These benefits depended on the possibility of accessing more complete and coherent patient information, regardless of where it was recorded, to support safe patient care. Additionally, in 1 use case, the core benefit was successful implementation of a new EHR through harmonizing data structures. An overview of the SNOMED CT core benefits is provided in Multimedia Appendix 3.

## Discussion

### Summary of Findings

This systematic review identified 17 articles in which SNOMED CT was implemented and used in a clinical context in EHRs or related clinical applications. We aimed to confirm whether research has developed to allow for a shift of focus from previously published reviews that described potential use toward studies documenting plausible benefits of SNOMED CT. We present findings related to clinical use purposes, use phases, and core benefits of SNOMED CT over the last 5 years of ongoing efforts. These review categories are based on previous research [3,5,13] that provided a strong starting point for this analysis.

The use purpose for SNOMED terminology based on previous research (Table 1) was identified in all the articles reviewed. As we evaluated the use cases, these categories served our research material well. The most applied use purpose category was SNOMED CT as the planned standard for EHRs or other related applications. Other frequently applied use categories were the goals of using SNOMED for retrieving and analyzing patient data or implementing the terminology to advance the coding of patient data. Only 2 of the articles in the review entailed proof of merit of EHR implementation as the use purpose category. Based on these results, the initial observation was that there might be a level of interconnectedness between the use purpose and use phase. To prove this, data on the maturity of EHR solutions are needed to research the possible interconnectedness of EHR use and SNOMED CT. Moreover, it might be relevant to analyze relationships between use purpose and use phase. This requires testing the categories and their possible relationships with different data sets.

Regarding the use phase results, all the reviewed articles included descriptions of the SNOMED CT use phase, although details of related contextual factors, such as clinical environment, varied. This hampered the assessment of the overall picture of the use phase. Considering the results, this may be a feature of this specific material, being typical of EHR-related use cases of SNOMED CT. Thus, in the future, it may prove fruitful to pay particular attention to the descriptions of these types of SNOMED CT use cases. We propose to describe the phase of use in a more structured and contextual manner. This kind of accuracy would increase the scalability of the use-case results. Lee et al [3] have already highlighted that only a few SNOMED CT implementation cases are being published in the scientific literature. Through the systematic investigation of previous theoretical work [3], and with time, more comparable scientific publications on SNOMED CT use cases in a clinical context could be published.

Based on our review, there is still little research evidence on the benefits for clinical use of SNOMED CT in EHR-related use cases. We identified the following frequently reported categories of core benefits: improvement of data quality and enabling a consistent way of indexing, storing, retrieving, and aggregating clinical data. Closely related to these benefits were improvement in quality of care—with the goal of achieving better patient safety—and, based on better data quality, enabling better continuity of care. Additionally, the review found individual remarks on improving the productivity of coding though automation or through terminological support for clinical users. Such tools had potential to increase user satisfaction, although there was evidence for a need to involve clinicians from different domains in development. Evidence of practical advancement may motivate various clinical specialties to become more involved in SNOMED CT development work from early on.

Although previous research has categorized the possible benefits of SNOMED CT [3,5], the core benefits in our review were summarized by the research team. By doing this, we aimed to describe the specific benefits of the EHR-related use of SNOMED CT. The objective is to highlight that the categorization applied in this study (Table 1) requires further testing with different data to assess its validity. The set of categories applied by our research team was not proven to be comprehensive or exhaustive. Therefore, in future research, it could prove relevant to carefully evaluate SNOMED CT use cases from the perspective of typical benefits. Additionally, it could be important to research what kinds of disadvantages, risks, and bottlenecks can be detected. By evaluating different use cases, it might be possible to extract the general success factors of clinical SNOMED CT implementation. Overall, the evaluation of SNOMED CT implementation requires more attention. As an example, the European large-scale implementation of SNOMED CT, which is being funded by the European Commission, could at the same time advance evaluation studies or require systematic evaluation as a part of the funding process.

Our review revealed that the EHR system or related software were poorly described in most of the articles, and specific descriptions of the EHRs were scattered due to a lack of uniform structure for such descriptions. This is clearly an issue that would require more attention in future use-case descriptions, given the fact that SNOMED CT is designed to support the use of EHRs. To describe the capabilities and overall maturity of the EHR system is core information in use case descriptions if the research result aims to benefit the clinical implementation process by avoiding previous obstacles and possible mistakes. We recognize that addressing potential mistakes does not mean that specific implementation experiences are universally generalizable, but that such implementation is required to be adapted to other clinical contexts. To promote the scalability of previous experiences, we suggest, for example, the application of the electronic medical record maturity model (EMRAM) in future use cases. EMRAM is a widely used tool developed by the Healthcare Information and Management Systems Society to measure the rate of adoption of EHR functions in health care settings. Its stages match the technological progress of the overall digitalization of the health care setting. Moreover, one possible starting point for future studies is to recognize specific use cases for software applications in clinical specialties, in which SNOMED CT would be valuable to accelerate and facilitate specific types of clinical implementations.

### Limitations

This systematic review has limitations that could affect the plausibility of the results. The methods and results of our systematic review are transparently reported in detail to allow readers to assess the trustworthiness and applicability of our findings [16]. However, even though the review’s methodological basis [16] is scientifically recommended, the varying levels of description in the research articles proved to be challenging; for example, the descriptions of background variables led to partial imprecision in the results. This especially affected the categories regarding the clinical context and type of EHR. The study’s risk of bias was carefully considered during the research process, and no assumptions were made about missing or unclear information from the studies.

We aimed to avoid missing key studies and to minimize bias by conducting a thorough and comprehensive literature search. However, our search entailed only one database, PubMed. Nevertheless, according to recent literature, PubMed can be used as a principal search system [34]. PubMed is well suited for evidence synthesis in the form of systematic reviews since it meets all necessary performance requirements, such as the formulation of queries, the correct interpretation of queries by the system, and the reproducibility of searches. At the same time, it is impossible to be certain that a system that has proven to be successful in specific tests will not fail under different circumstances [34,35]. Moreover, not all SNOMED CT implementation projects are published in the scientific literature [3]. Thus, it is important to recognize that relevant information on clinical use cases may be found in different types of literature.

### Conclusions

This literature review demonstrates that systematic reviews are relevant to the development of an understanding of SNOMED CT use and its possible benefits in further facilitating multi-professional, clinically driven implementations by summarizing essential findings based on evidence-based results. Clinical use cases are needed to promote the scalability of review results. To achieve the best out-of-use case reports, more emphasis should be placed on describing the contextual factors, such as the electronic health care system currently in use and the use of previous frameworks, to allow the comparability of results. Regarding future research, although other systematic reviews have addressed similar questions to ours, this review is necessary to shift the focus onto more clinically grounded implementation outcomes and benefits of the use of SNOMED CT. Generally, further research evidence is still needed to determine how exactly SNOMED CT benefits clinical care and patient information quality.

## Appendix

Multimedia Appendix 1 Outline of the review protocol applied in this research.

Multimedia Appendix 2 Characteristics of the publications.

Multimedia Appendix 3 Overview of the results.

## Abbreviations

EHR: electronic health record
EMRAM: Electronic Medical Record Adoption Model
SNOMED CT: Systematized Nomenclature of Medicine–Clinical Terminology
