# Supplementary material for: Systematized Nomenclature of Medicine–Clinical Terminology (SNOMED CT) Clinical Use Cases in the Context of Electronic Health Record Systems: Systematic Literature Review
Source: JMIR Med Inform. 2023 Feb 6;11:e43750. doi: 10.2196/43750 (PMC9941898; doi:10.2196/43750)
Supplement: Multimedia Appendix 1 [file medinform_v11i1e43750_app1.docx]

Multimedia Appendix 1. Outline of the review protocol applied in this research

| 1 | Defining the research questions |
| --- | --- |
| 2 | Defining the database and search strategy based on the research questions |
| 3 | Conducting a test search and updating the search strategy accordingly |
| 4 | Conducting the searches, removing duplicates, and, within the team, agreeing upon how to save and manage the search results |
| 5 | Defining exclusion and inclusion criteria |
| 6 | Excluding by two researchers reading all articles’ headings and abstracts and agreeing upon the exclusion results within the research team |
| 7 | Inclusion by two researchers reading the remaining articles’ full texts and discussing the results within the team |
| 8 | Final inclusion and consensus of remaining articles based on the research question and agreement within the research team |
| 9 | Extracting and documenting the information from the review articles by two researchers using a template defined and refined by the team according to the research questions |
| 10 | Finalizing the review analysis and generating/writing the review report |
