# Supplementary material for: Systematized Nomenclature of Medicine–Clinical Terminology (SNOMED CT) Clinical Use Cases in the Context of Electronic Health Record Systems: Systematic Literature Review
Source: JMIR Med Inform. 2023 Feb 6;11:e43750. doi: 10.2196/43750 (PMC9941898; doi:10.2196/43750)
Supplement: Multimedia Appendix 2 [file medinform_v11i1e43750_app2.docx]

Multimedia Appendix 2: Characteristics of the publications

| Reference | Year of publication | Country | Journal |
| --- | --- | --- | --- |
| [17] | 2020 | Canada | Comput Methods Programs Biomed |
| [18] | 2021 | Denmark | Stud Health Technol Inform |
| [19] | 2022 | UK | Orphanet J Rare Dis |
| [20] | 2020 | USA | AMIA Jt Summits Transl Sci Proc |
| [21] | 2021 | Switzerland | JMIR Med Inform |
| [22] | 2019 | USA | BMJ Health Care Inform |
| [23] | 2021 | UK | Artif Intell Med |
| [24] | 2021 | UK | Health Technol (Berl) |
| [25] | 2022 | Australia | Clin Rheumatol |
| [26] | 2018 | Australia | AMIA Annu Symp Proc |
| [27] | 2021 | UK | JMIR Med Inform |
| [28] | 2021 | Spain | Stud Health Technol Inform |
| [29] | 2020 | Republic of Korea | J Med Internet Res |
| [30] | 2019 | Germany | Stud Health Technol Inform |
| [31] | 2018 | Spain | Int J Environ Res Public Health |
| [32] | 2017 | UK | Future Healthc J |
| [33] | 2018 | USA | Appl Clin Inform |
