# Supplementary material for: Systematized Nomenclature of Medicine–Clinical Terminology (SNOMED CT) Clinical Use Cases in the Context of Electronic Health Record Systems: Systematic Literature Review
Source: JMIR Med Inform. 2023 Feb 6;11:e43750. doi: 10.2196/43750 (PMC9941898; doi:10.2196/43750)
Supplement: Multimedia Appendix 3 [file medinform_v11i1e43750_app3.docx]

Multimedia Appendix 3: Overview of the results

| Reference | Clinical use context | EHR | Users | SNOMED CT use purpose | SNOMED CT use phase | Core benefits from EHR use case |
| --- | --- | --- | --- | --- | --- | --- |
| [17] | Neurology (stroke unit) | System integrated to EHR | Not specified | Planned standard for EHR | In use, proof of merit | To improve quality of care (patient safety) |
| [18] | Hospital emergency room (pre-hospital unit) | Pre-hospital health record not integrated to hospital EHR | Clinicians, emergency nurses | Planned standard for EHR | In development in the pre-hospital health record, in use in hospital EHR | To improve continuity of care (possible implications for care quality, patient safety) |
| [19] | Rare diseases | Integrated software for clinical use | General practitioners | Planned standard for EHR | In pilot | Enabling a consistent way of indexing, storing, retrieving, and aggregating clinical data |
| [20] | Pediatric | Pediatric learning hospital EHR | Not specified | To retrieve or analyze patient data | In use | To improve data quality |
| [21] | Diagnostics | University hospital clinical system (EHR) | Clinical domain experts, clinical coders | Planned standard for a clinical application | In implementation | To improve data quality |
| [22] | Primary care (problem list) | Primary care EHR | Physician, advanced practice nurses | Planned standard for a clinical application | In implementation | Enabling a consistent way of indexing (especially diagnosis data) |
| [23] | Several (secondary and tertiary care) | Mature hospital EHR | Clinicians, medical students (in the study) | To retrieve and analyze patient data (data extraction across multiple hospitals) | In use | To improve data quality |
| [24] | Primary care | Primary care EHR | General practitioners | To prove merit of SNOMED CT | In use | To improve data quality (to improve care) |
| [25] | General medicine Rheumatology | Local EHR system | Not specified | Data extraction (used to classify or code in a study) | In use | Enabling a consistent way of indexing, storing, retrieving, and aggregating clinical data |
| [26] | Not specified | Hospital EHR | Clinical coders | Development for automated coding | In development | To improve data quality and productivity of coding |
| [27] | Secondary and tertiary care | Hospital central EHR | Clinicians, developers | Planned standard for EHR | In implementation | To improve data quality and successful implementation |
| [28] | Not specified | Hospital EHR | Clinical domain experts, terminologists, computer scientists | Planned standard for EHR | In implementation | To improve patient care (possible implications for patient safety), to improve data quality for patient care and for research |
| [29] | Oncology  Tertiary care | Hospital EHR | Medical informatics expert, nurse | Data extraction | In development | Enabling a consistent way of retrieving patient information |
| [30] | Pulmonology | Outpatient and inpatient EHRs | General practitioners, clinicians | Planned standard for EHR | In development | To improve quality of care (implications for patient safety) |
| [31] | Cardiology | Hospital EHR | Not specified | Planned standard for EHR | In development | To improve quality of care (implications for patient safety) |
| [32] | Neurology | Centralized EHR with web-interface | Clinicians | Prove of merit of SNOMED CT | After implementation evaluation | To improve data quality |
| [33] | Not specified | EHR | Clinicians, clinical coders, researchers, physician informaticians | Planned standard for EHR | In development | Enabling a consistent way of indexing, storing, retrieving and aggregating clinical data |
